# Unruptured aneurysmal clipping complicated by delayed and refractory vasospasm: case report

**DOI:** 10.1186/s12883-020-01925-8

**Published:** 2020-09-12

**Authors:** Crina M. Peterson, Sahitya S. Podila, Tarun Girotra

**Affiliations:** 1 School of Medicine, University of New Mexico, Albuquerque, NM USA; 2 Department of Neurology, University of New Mexico, Albuquerque, NM USA

**Keywords:** Unruptured intracranial aneurysms, Cerebral vasospasm, Aneurysmal clipping, Case report

## Abstract

**Background:**

Delayed symptomatic vasospasm is a rare complication following clipping of an unruptured intracranial saccular aneurysm. There have been ten reported cases of delayed symptomatic vasospasm and only two of these occurred after 2 weeks from initial intervention. Our case is the first to document the refractory nature of such vasospasm despite aggressive first line therapy.

**Case presentation:**

Here, we present a 67-year-old female who had surgical clipping of a 10x7mm right middle cerebral artery (MCA) bifurcation aneurysm. Her surgery and initial postoperative course were uncomplicated, but she presented with acute left hemiparesis, dysarthria, headache and vomiting on post-op day 29 secondary to vasospasm of M2. She was initially stabilized with intra-arterial verapamil then managed with volume expansion, permissive hypertension, and nimodipine. She developed recurrent vasospasm of M2 the following day and was again treated with intra-arterial verapamil. Magnetic resonance imaging (MRI) brain showed an infarction involving the right basal ganglia, frontal lobe, and parietal lobe and her hospital course was complicated by super-refractory status epilepticus. At her follow up appointment she displayed continued left lower extremity weakness, left visual field defect, and left-sided neglect.

**Conclusions:**

Overall, cerebral vasospasms associated with unruptured aneurysms remain rare complications and are not often monitored for after initial recovery. Reviewing the documented cases highlights the unpredictability of when these events occur with our current knowledge. Current hypotheses for the mechanisms responsible for delayed and refractory vasospasms include: blood-derived breakdown products, mechanically induced vasospastic responses, and delayed reactions from the trigemino-cerebrovascular system (TCVS). The uncertainly of these events warrants further research and supports a strong argument for monitoring patients with initial surgical clipping up to a month out from their initial procedure.

## Background

Intracranial saccular aneurysms are described as an outpouching of the tunica intima and adventitia of an arterial wall caused by collagen deficiency in the internal elastic lamina and breakdown of the tunica media [1]. They are typically found in proximal branch points of the circle of Willis, are usually small and asymptomatic, and have an overall prevalence of approximately 1.8–3.2% [2, 3]. Ruptured aneurysms are the cause of approximately 83–85% of subarachnoid hemorrhages [4, 5] and may also lead to intraparenchymal hemorrhage, subdural hematoma, or intraventricular hemorrhage. Aneurysm coiling and clipping are the most common procedures used to prevent rupture of aneurysms and are often done in the case of large or enlarging aneurysms [6]. Complications for these procedures include intracerebral hemorrhage, postoperative stroke, hydrocephalus, status epilepticus, cardiac complications, pulmonary complications, systemic infection, and acute renal failure [7]. A recent meta-analysis reported that ischemic complications occur in 2.52% of clipped unruptured aneurysms within the first 30 days following surgery [8]. Delayed vasospasm remains a rare cause of ischemic stroke following unruptured aneurysm clipping and has been reported to occur 5–28 days following surgery [9]. Vasospasm that occurs earlier in the postoperative period most commonly develops within hours of surgery, although no absolute definition has been made to distinguish early and delayed vasospasm [10]. The mechanism for early vasospasm is thought to be due to mechanical stress from surgical manipulation; however, there is no current consensus on a mechanism explaining delayed vasospasm [9]. Causes of delayed injury due to other etiologies are often ruled out before delayed vasospasm is considered; they include increased edema, rebleeding of remnant, hydrocephalus, infection, hyponatremia, hypoxemia, cortical spreading depressions [11]. Here we report a case of a patient who developed complications of a delayed refractory vasospasm 29 days after clipping of a saccular aneurysm at the right MCA bifurcation. To our knowledge there are currently only two reported cases of delayed vasospasms after 2 weeks from initial intervention (Table 1) [9, 10, 12–17] but ours is the first to document the refractory nature of such vasospasm despite aggressive first line therapy.

**Table 1:** Previous case reports involving symptomatic vasospasm post-clipping of unruptured intracranial aneurysms

Reference	Patient	Aneurysm location	Symptoms	Spasm POD	Treatments	Deficits
(Harrop et al., 2009) [12]	38/F	Left internal carotid artery bifurcation	Aphasia, right hemiparesis	7	Hypertension, hypervolemia, hemodilution (Triple H)	None
(Kitazawa et al., 2005) [13]	53/F	Left paraclinoid carotid	Aphasia	9	Triple H	None
(Bloomfield & Sonntag, 1985) [14]	54/F	Right MCA bifurcation	Left hemiparesis	9	Hypervolemia, dexamethasone	Weakness
(Ou et al., 2017) [9]	50/M	Right M2	Headache, aphasia, left hemiparesis	10	Nimodipine, hypervolemia, antiplatelet, hyperbaric O2	Weakness
(Yang et al., 2014) [15]	61/F	Left MCA bifurcation	Aphasia, mental status changes	10	Nicardipine, hydration, antiplatelet	Partial aphasia
(Hashimoto et al., 2016) [16]	62/F	Left internal carotid artery- posterior communicating artery	Headache, aphasia, right hemiplegia	11	Hypervolemia, antiplatelet	Acalcula, paraphasia
(Kitazawa et al., 2005) [13]	21/F	Left paraclinoid carotid	Aphasia, Gerstmann syndrome	12	Papaverine, hyperbaric O2, Triple H	None
(Campe et al., 2019) [17]	69/F	Right MCA bifurcation	Aphasia, left hemiparesis	12	Nimodipine, antiplatelet	None
(Yang et al., 2014) [15]	41/F	Left internal carotid bifurcation	Aphasia, right facial numbness	28	Nicardipine, hydration, antiplatelet	Partial aphasia
(Paolini et al., 2005) [10]	47/F	Right MCA bifurcation	Left hemiparesis	28	Hypervolemia, antiplatelet	None

## Case presentation

**Fig. 1:**
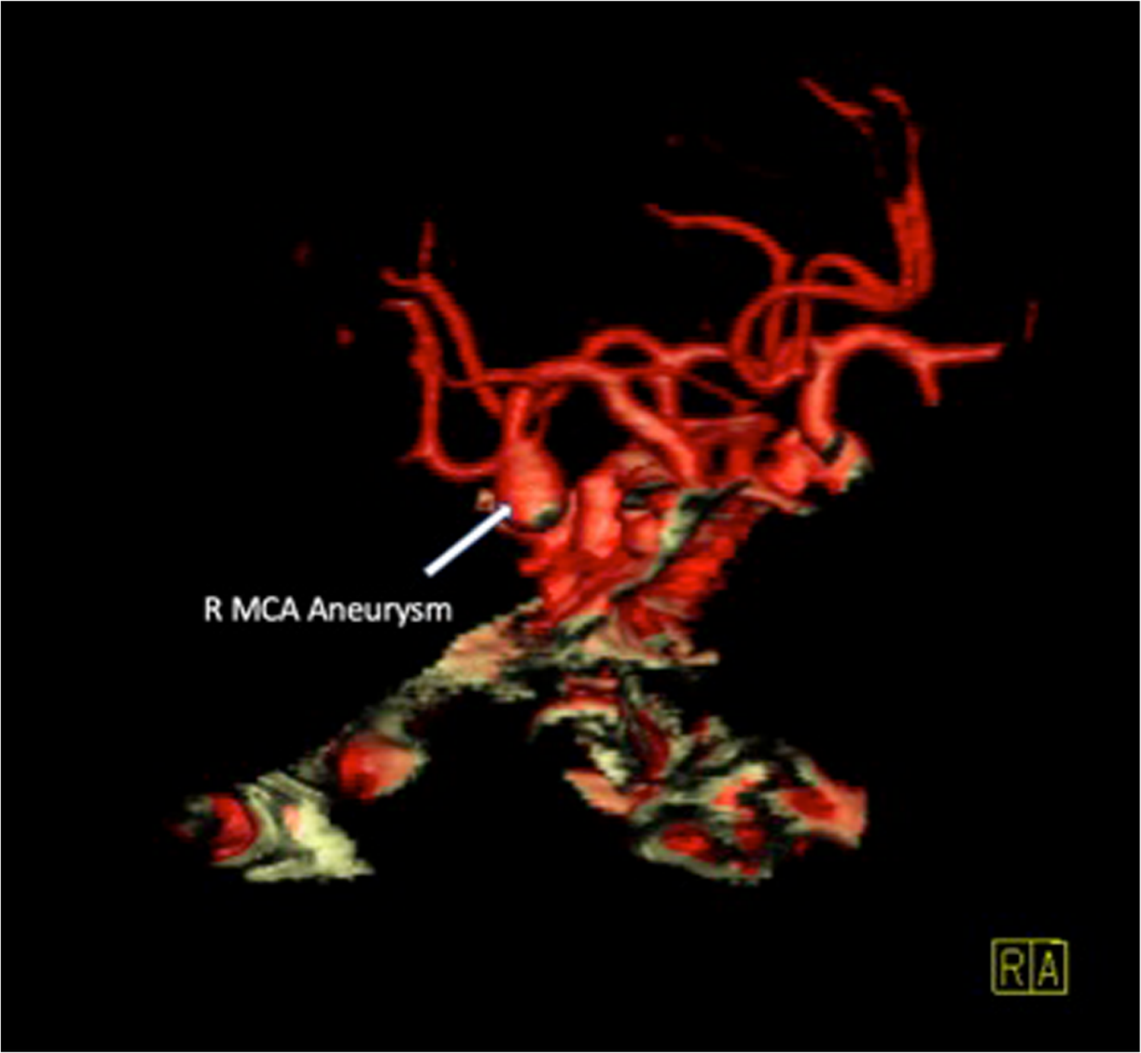
3D reconstruction of pre-operative CT angiogram. A 10 × 7 mm right MCA bifurcation aneurysm (white arrow) is shown

A 67-year-old female with multiple comorbidities presented to the emergency department following a syncopal episode. She denied any other neurological symptoms, neurological exam was normal, and a computed tomography angiography (CTA) head demonstrated an incidental 8 × 6 mm aneurysm at the right MCA bifurcation. She opted for surveillance initially, but 1 year later, CTA head showed that the aneurysm had grown to 10x7mm (Fig. 1). At this time, after discussion with vascular neurosurgery, patient opted for elective clipping of the right MCA bifurcation aneurysm.

The patient was sedated with general anesthesia, and a right pterional craniotomy was performed. Microdissection was carried out from distal to proximal to open the sylvian fissure exposing the proximal MCA and a temporary clip was applied to the M1 to gain proximal control. The bifurcation was dissected, and a 9 mm straight permanent aneurysm clip was placed at the neck of the aneurysm. Intraoperative Doppler had shown appropriate flow of the M1 and M2 both prior to clipping and following clipping. A small area of aneurysm residual was appreciated posteriorly, and a 3-mm fenestrated clip was used to safely close off the remaining aneurysm. A spinal needle was then inserted into the aneurysm to decompress it successfully, and all layers were then closed. Electrophysiological monitoring with motor evoked potential remained stable from baseline throughout the entirety of the procedure. The patient tolerated the procedure well, and she was transferred to the Neuro ICU in stable condition and finally discharged on post-operative day 3 without any complications.

**Fig. 2:**
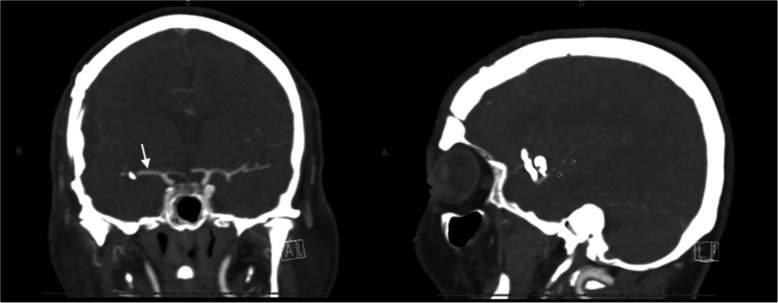
CT angiogram on Day-0 of acute left hemiparesis. Left (white arrow) showing a patent M-1 segment of the right MCA and Right (Dashed arrows) showing spastic M2 segment of the right MCA

**Fig. 3:**
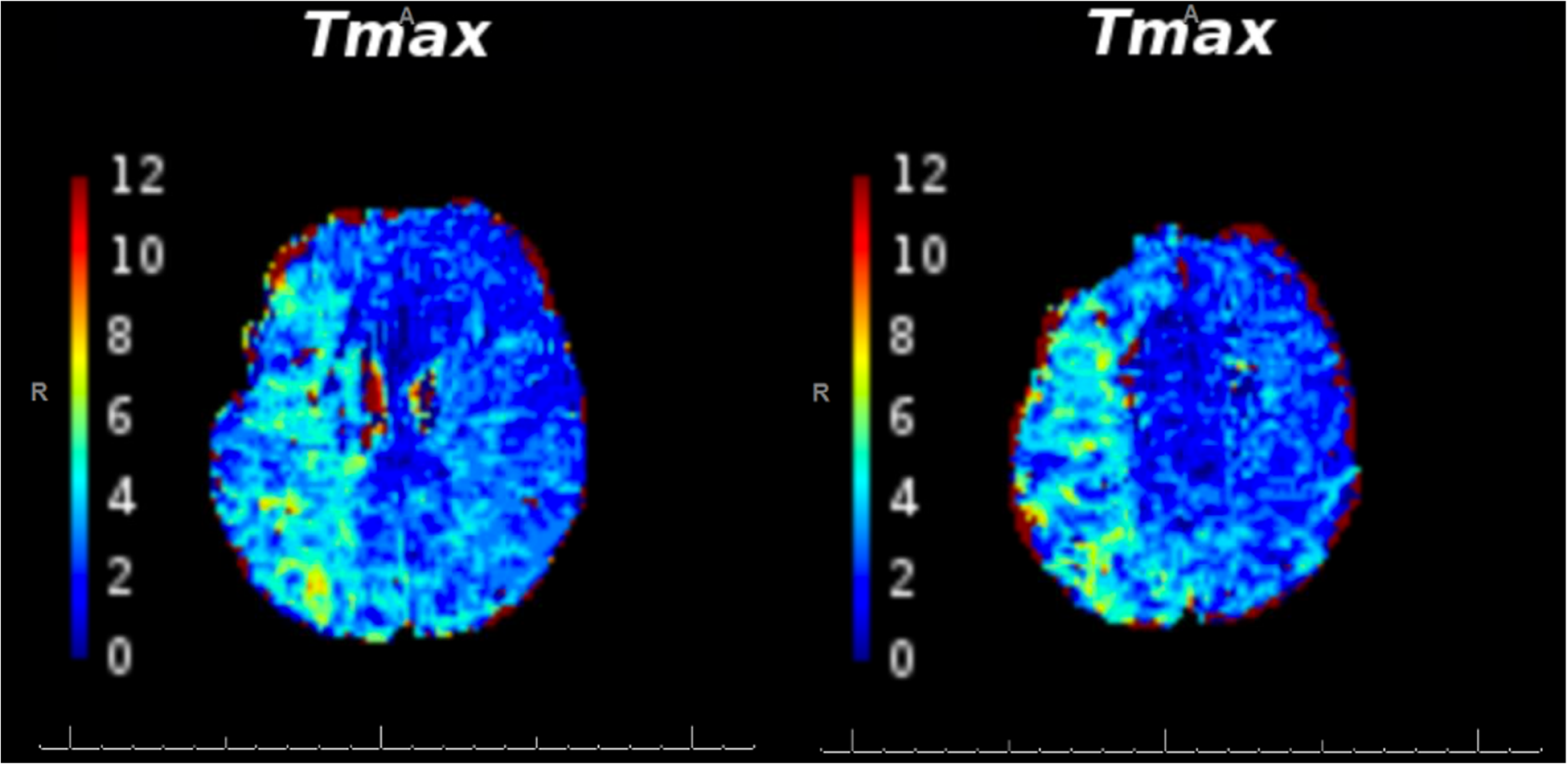
MR Perfusion study on Day-0 of acute hemiparesis. Slower flow in the right MCA territory when compared to the left MCA territory is shown

At 29 days post-op, the patient presented with acute left hemiparesis, dysarthria, headache and vomiting. Vascular studies with CTA head showed significant narrowing of M2 distal to surgical clip (Fig. 2). Emergent MR perfusion study showed slow flow throughout the right MCA territory (Fig. 3) but no irreversible infarction was clear at this point.

**Fig. 4:**
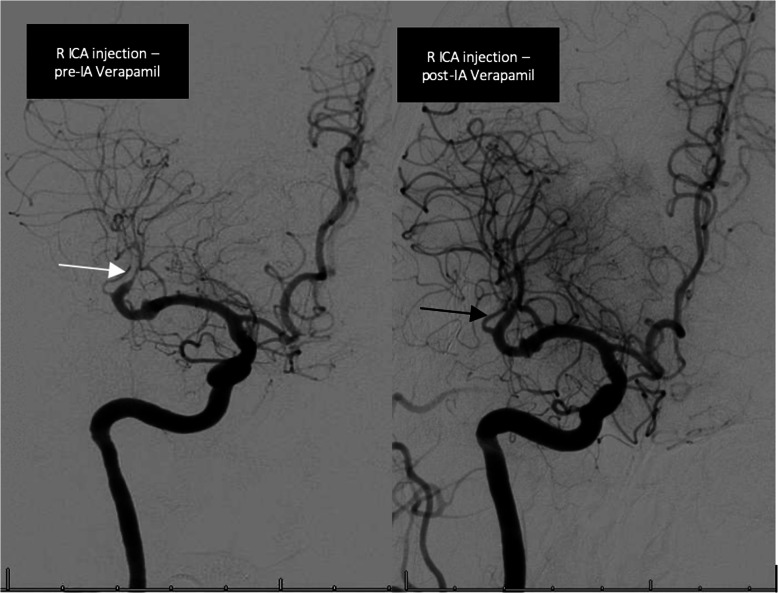
Catheter angiogram on Day-0 of acute right hemiparesis. Left (white arrow) representing the vasospasm in M-2 segment of the right MCA. Right (black arrow) representing resolution of vasospasm after intra-arterial verapamil

Patient underwent an emergent catheter angiogram (Fig. 4), during which she received 20 mg intra-arterial verapamil with resolution of her vasospasm (Fig. 4), and improvement in her neurological exam. She was admitted to the neurocritical care unit and managed by volume expansion and permissive hypertension. She was also started on aspirin 81 mg daily.

**Fig. 5:**
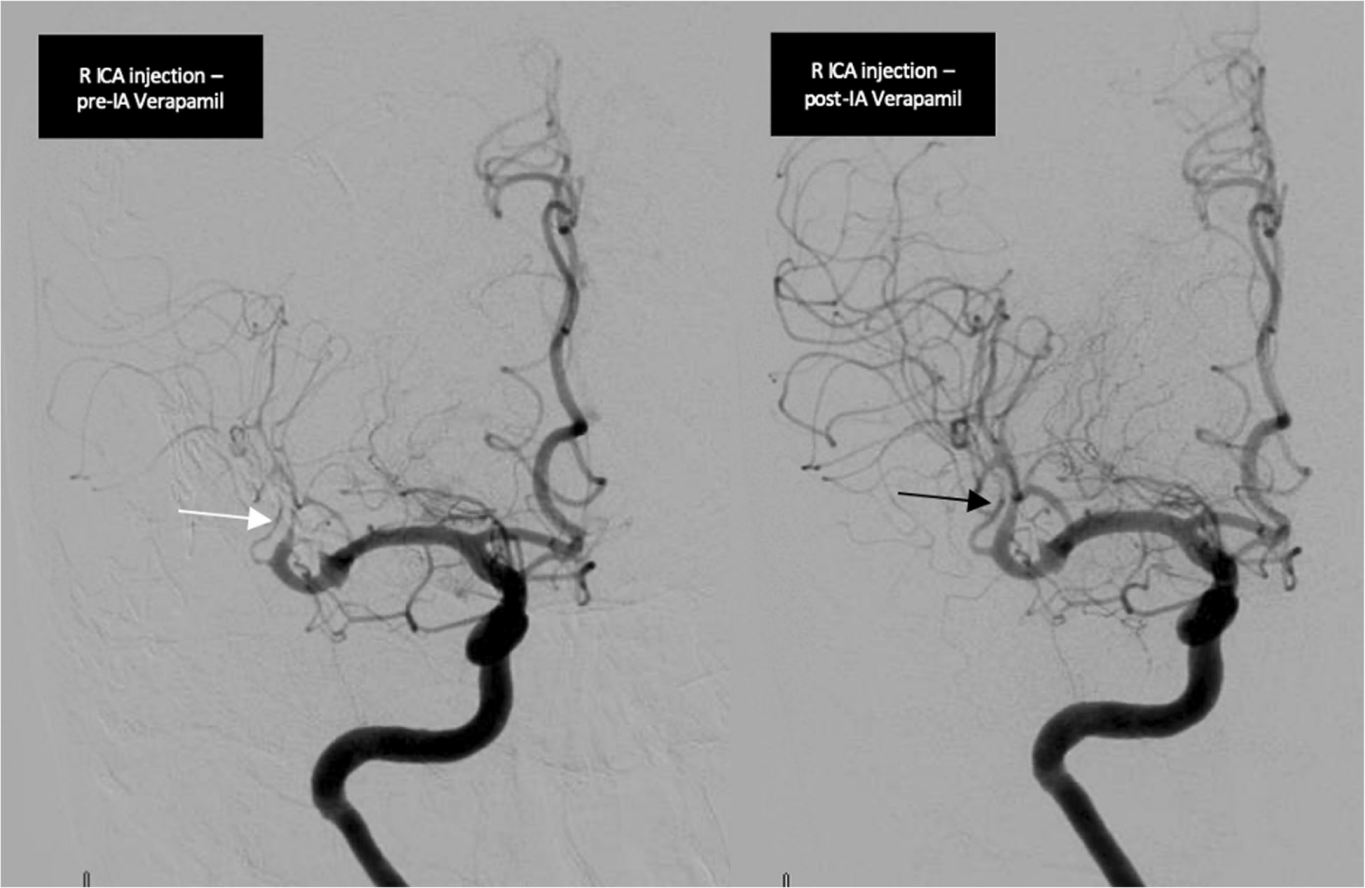
Catheter angiogram on Day-1 of acute right hemiparesis. Left (white arrow) representing the recurrence of vasospasm in M-2 segment of the right MCA. Right (black arrow) representing improvement of vasospasm after intra-arterial verapamil

**Fig. 6:**
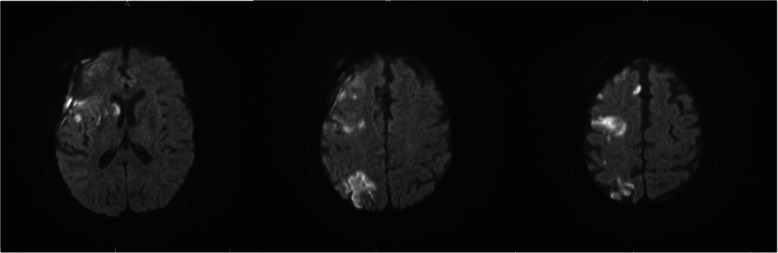
Diffusion weighted imaging sequence of MRI brain on Day-1. Acute ischemic infarct involving the right hemisphere is shown

Twenty-four hours later, her neurological exam deteriorated. Emergent repeat catheter angiogram revealed recurrent vasospasm in the same vessel (Fig. 5). She received 20 mg intra-arterial verapamil again with improvement in vasospasm (Fig. 5), but unfortunately her MRI brain showed an infarction involving the right basal ganglia, frontal lobe, and parietal lobe (Fig. 6).

Her hospital course was further complicated by super-refractory status epilepticus with epileptic discharges originating from the right fronto-temporal region. Seizures were eventually controlled with multiple anti-epileptic medications, and she also finished a 21-day course of nimodipine. Follow-up CTA head on day 15 of admission revealed persistent absence of vasospasm. She was discharged to a skilled nursing facility after a prolonged hospital course.

The patient’s functional status was moderately improved at her follow up appointment 2.5 months post-op. MRI showed normal evolution of ischemic stroke, with no acute findings. She was able to walk but continued to have left lower extremity weakness, left visual field defect, and left-sided neglect. She had mild improvement in mental status but had difficulty with memory. She did not have any concerns for continued seizures or stroke-like symptoms.

## Discussion and conclusions

Here, we presented a patient who was initially treated for an MCA bifurcation aneurysm by surgical clipping but later presented with delayed vasospasm 29 days post-op. The patient additionally had a refractory vasospasm in the same location despite initial intra-arterial verapamil during her hospitalization. Overall, she received two courses of 20 mg intra-arterial verapamil and a 21-day course of nimodipine. Unfortunately, despite initial success with surgical clipping, she did suffer long-term consequences of left lower extremity weakness, left visual field defect with left-sided neglect, and some mental status changes due to her delayed vasospasm.

Besides our case, to our knowledge there are only two described cases of delayed vasospasms greater than 2 weeks post-surgically. First, Paolini et al. describes a case of a 47-year-old woman who developed a symptomatic vasospasm 28 days after clipping of a MCA aneurysm [10]. The patient was stable and discharged post-surgically with no complications, and 28 days later a CTA revealed vasospasm of the distal M1 segment. She was treated with volume expansion and anti-platelet therapy and recovered to baseline within 12 h [10]. The second incidence of a severely delayed vasospasm was reported by Yang and colleagues. They described a 41-year-old woman who was worked up for a non-specific headache and found to have an unruptured intracranial aneurysm in the left internal carotid artery bifurcation [15]. Her post-operative course was uneventful; however, on post-op day 28 she presented with right facial numbness along with decreased strength of hand and aphasia and was found to have a vasospasm of the left distal internal carotid artery in the A1 and M1 segments [15]. This patient received a chemical angioplasty with nicardipine, along with volume expansion and anti-platelet therapy. As the previous patient, she returned to baseline and had no recurrence.

The mechanism underlying post-surgical vasospasms, and especially, delayed vasospasm is poorly understood [18]. Among the few reported cases with delayed vasospasm, latency ranges from 5 to 28 days, now 29 with this present case. Additionally, most reported cases describe the distribution of vasospasm as regional, which suggests that factors in proximity of an aneurysm contribute to later complications [10]. It seems that another delineation should exist between delayed and severely delayed, as hypotheses for vasospasms seem to be time dependent. One older hypothesis postulates that blood-derived breakdown products are responsible for vasospastic events, as there seems to be a relationship between subarachnoid blood and the incidence of vasospasm [19, 20]. The incidence of these events in patients with unruptured aneurysms seems to challenge this hypothesis. However, the aneurysm itself may be a source of blood breakdown products, not just subarachnoid cisterns, and this could account for the delay between surgery and onset [21]. Over time, multiple studies have documented that endothelial-derived factors to be spasmogenic, regardless of presence of subarachnoid blood [22, 23]. Surgical manipulation can result in minimal bleeding, but this is usually well-irrigated and does not seem to be correlated to vasospastic events. Another theory focuses on a mechanically induced vasospastic response as a result of manipulation of the Sylvian fissure and MCA branches, but vasospasms in this circumstance are modest and occur within 7 days of surgery [24]. More recently, some have focused on disruption of the TCVS, a nerve network surrounding arteries of the circle of Willis, as a source of vasospastic events [25, 26]. Experimental studies have demonstrated an association between post-subarachnoid hemorrhage (SAH) vasospasm and TCVS-mediated reflexes leading to vasodilatory peptides [26]. Finally, an observation that has yet to be described is that besides one patient, the others identified in our table have all been female. The vasodilatory properties of estrogen are well established, but it would be interesting to assess whether other sex-specific factors could attribute to these findings.

Currently, there are few management options for patients that experience delayed vasospasm. Most include a combination of volume expansion and permissive hypertension with antiplatelet therapy and/or vasodilation with intra-arterial therapy and/or nimodipine. It is unclear what dosage of aspirin was used for antiplatelet therapy in some cases. Our patient received 81 mg daily, but an increased dose would have to be balanced with concern for bleeding. Besides our patient, three other reported cases used intra-arterial vasodilators including nicardipine and papaverine [13, 15]. After verapamil administration, our patient improved clinically on day of presentation, so it remains feasible that this can successfully be used as a rescue therapy in severe cases. Additionally, Albanese and colleagues have shown that prolonged verapamil administration is safe and effective in medically refractory vasospasm [27]. Further efforts should be dedicated to investigating the optimal dose and duration of verapamil treatment in both initial and refractory vasospasm.

There are no effective animal models to study the temporal or physiologic nature of delayed cerebral vasospasms. Kumagi and colleagues at Tokyo General Hospital developed a recent rat model, with induction of SAH followed by unilateral common carotid artery occlusion, which mimics early cerebral hypoperfusion and leads to delayed brain injury without significant mortality [28]. This will allow for controlled observation of developing vasospasms at different timepoints and timed interventions with currently approved treatments such as vasodilators and intra-arterial angioplasties. Case reports such as these are important in order to provide valuable hypotheses as animal models are developed.

Cerebral vasospasms associated with unruptured aneurysms remain rare complications; however, there may exist unreported cases in addition to cases with vasospasms after flow-diverting procedures. The strengths of this report include the first mention of a refractory vasospasm in addition to a delayed event after aggressive therapy and a thorough review of literature. Case reports such as these can bring to light rare, although important, clinical observations that need to be addressed for improved patient outcomes. However, there are several limitations common to all case reports. The event of a delayed vasospasm is still rare clinically, and significant epidemiological data does not exist. Additionally, causal relations cannot be identified beyond suggesting and supporting certain hypotheses. Finally, this patient received care from various clinicians, and the report was written retrospectively, missing the opportunity to control for variables due to care.

It is likely that different mechanisms account for delayed vasospasms compared to early brain injuries seen in patients after clipping of aneurysms and further investigations are necessary to elucidate these. Because these events occur and can be devastating, ensuring close monitoring of neurological status up to several weeks after an event will be paramount to identifying delayed and refractory cases of vasospasm.

## Data Availability

Data sharing is not applicable to this article as no datasets were generated or analyzed during the current study.

## Abbreviations

MCA: Middle cerebral artery
MRI: Magnetic resonance imaging
TCVS: Trigemino-cerebrovascular system
CTA: Computed tomography angiography
SAH: Subarachnoid hemorrhage

## References

[1] Keedy A (2006). An overview of intracranial aneurysms. Mcgill J Med.

[2] Vernooij MW, Ikram MA, Tanghe HL, Vincent AJ, Hofman A, Krestin GP (2007). Incidental findings on brain MRI in the general population. N Engl J Med.

[3] Vlak MH, Algra A, Brandenburg R, Rinkel GJ (2011). Prevalence of unruptured intracranial aneurysms, with emphasis on sex, age, comorbidity, country, and time period: a systematic review and meta-analysis. Lancet Neurol.

[4] Kassell NF, Torner JC, Jane JA, Haley EC, Adams HP (1990). The international cooperative study on the timing of aneurysm surgery. Part 2: surgical results. J Neurosurg.

[5] van Gijn J, Rinkel GJ (2001). Subarachnoid haemorrhage: diagnosis, causes and management. Brain..

[6] Thompson BG, Brown RD, Amin-Hanjani S, Broderick JP, Cockroft KM, Connolly ES (2015). Guidelines for the Management of Patients with Unruptured Intracranial Aneurysms: a guideline for healthcare professionals from the American Heart Association/American Stroke Association. Stroke..

[7] Alshekhlee A, Mehta S, Edgell RC, Vora N, Feen E, Mohammadi A (2010). Hospital mortality and complications of electively clipped or coiled unruptured intracranial aneurysm. Stroke..

[8] Algra AM, Lindgren A, Vergouwen MDI (2019). Procedural clinical complications, case-fatality risks, and risk factors in endovascular and neurosurgical treatment of Unruptured intracranial aneurysms: a systematic review and meta-analysis. JAMA Neurol.

[9] Ou C, Chen Y, Wang S, Mo J, Hu W (2017). Case report delayed symptomatic vasospasm after clipping of an unruptured intracranial aneurysm: case report and literature review. Int J Clin Exp Med.

[10] Paolini S, Kanaan Y, Wagenbach A, Fraser K, Lanzino G (2005). Cerebral vasospasm in patients with unruptured intracranial aneurysms. Acta Neurochir.

[11] Findlay JM, Nisar J, Darsaut T (2016). Cerebral Vasospasm: A Review. Can J Neurol Sci.

[12] Harrop MD, Rosenwasser MD, Robert H (2009). Symptomatic cerebral vasospasm after surgical ligation of Unruptured aneurysms. JHN J.

[13] Kitazawa K, Hongo K, Tanaka Y, Oikawa S, Kyoshima K, Kobayashi S (2005). Postoperative vasospasm of unruptured paraclinoid carotid aneurysms: analysis of 30 cases. J Clin Neurosci.

[14] Bloomfield SM, Sonntag VK (1985). Delayed cerebral vasospasm after uncomplicated operation on an unruptured aneurysm: case report. Neurosurgery..

[15] Yang K, Ahn JS, Park JC, Kwon DH, Kwun BD (2014). Clinical and angiographical delayed cerebral vasospasms after uncomplicated surgical clipping of unruptured intracranial aneurysms: illustrated review and two case reports added. Turkish Neurosurg.

[16] Hashimoto H, Kameda M, Yasuhara T, Date I (2016). A case of unexpected symptomatic vasospasm after clipping surgery for an Unruptured intracranial aneurysm. J Stroke Cerebrovasc Dis.

[17] Campe C, Neumann J, Sandalcioglu IE, Rashidi A, Luchtmann M (2019). Vasospasm and delayed cerebral ischemia after uneventful clipping of an unruptured intracranial aneurysm - a case report. BMC Neurol.

[18] Hurth H, Birkenhauer U, Steiner J, Schlak D, Hennersdorf F, Ebner FH (2020). Delayed cerebral ischemia in patients with aneurysmal subarachnoid Hemorrhage - serum D-dimer and C-reactive protein as early markers. J Stroke Cerebrovasc Dis.

[19] Asano T, Tanishima T, Sasaki T, Sano K (1980). Possible participation of free radical reactions initiated by clot lysis in the pathogenesis of vasospasm after subarachnoid hemorrhage. *Cerebral Arterial Spasm*.

[20] Fisher CM, Kistler JP, Davis JM (1980). Relation of cerebral vasospasm to subarachnoid hemorrhage visualized by computerized tomographic scanning. Neurosurgery..

[21] DeLong WB (1980). Severe vasospasm with an unruptured aneurysm. Neurosurgery..

[22] Nishizawa S, Chen D, Yokoyama T, Yokota N, Otha S (2000). Endothelin-1 initiates the development of vasospasm after subarachnoid haemorrhage through protein kinase C activation, but does not contribute to prolonged vasospasm. Acta Neurochir.

[23] Zimmermann M, Seifert V (1998). Endothelin and subarachnoid hemorrhage: an overview. Neurosurgery..

[24] Schaller C, Klemm E, Haun D, Schramm J, Meyer B (2002). The transsylvian approach is “minimally invasive” but not “atraumatic”. Neurosurgery..

[25] Büki A, Horváth Z, Kalló I, Liposits Z, Lengvári I, Dóczi TP (1999). Peptidergic innervation of human cerebral blood vessels and saccular aneurysms. Acta Neuropathol.

[26] Edvinsson L, Juul R, Jansen I (1994). Perivascular neuropeptides (NPY, VIP, CGRP and SP) in human brain vessels after subarachnoid haemorrhage. Acta Neurol Scand.

[27] Albanese E, Russo A, Quiroga M, Willis RN, Mericle RA, Ulm AJ (2010). Ultrahigh-dose intraarterial infusion of verapamil through an indwelling microcatheter for medically refractory severe vasospasm: initial experience, Clinical article. J Neurosurg.

[28] Kumagai K, Tomiyama A, Takeuchi S, Otani N, Fujita M, Fujii K (2019). New endovascular perforation subarachnoid hemorrhage model for investigating the mechanisms of delayed brain injury. J Neurosurg.

